# Circuit Training Improves the Levels of β-Amyloid and Brain-Derived Neurotrophic Factor Related to Cognitive Impairment Risk Factors in Obese Elderly Korean Women

**DOI:** 10.3390/jcm13030799

**Published:** 2024-01-30

**Authors:** Duwang Kang, Suhan Koh, Taekyu Kim, Eadric Bressel, Doyeon Kim

**Affiliations:** 1 Department of Physical Education, Pusan National University, Busan 46241, Republic of Korea; 2 Department of Kinesiology and Health Science, Utah State University, Logan, UT 84322, USA

**Keywords:** circuit training, β-amyloid, brain-derived neurotrophic factor, cognitive function, obese elderly women

## Abstract

**Background**: The purpose of this study was to investigate the effect of circuit training on β-amyloid, BDNF, and cognitive function in untrained obese elderly Korean women. **Methods**: The subjects for the study were aged 65–70 years and were each assigned to a circuit training group (EG, *n* = 12) or a control group (CG, *n* = 11). The 60 min combined exercise was performed 3 times per week for 16 weeks. The exercise intensity was progressively increased from a 40% heart rate reserve to a 70% heart rate reserve. The test data were analyzed using a paired *t*-test, an independent *t*-test, and a two-way repeated measures ANOVA, and an alpha level of 0.05 was set for all tests of significance. **Results**: Group-by-time interaction effects were observed for β-amyloid (*p* < 0.05), brain-derived neurotrophic factor (*p* < 0.01), and cognitive function (*p* < 0.05). Within the exercise group, significant differences were found in β-amyloid (*p* < 0.05), brain-derived neurotrophic factor (*p* < 0.001), and cognitive function (*p* < 0.05) when comparing across different time points. Additionally, there were statistically significant differences between groups in post-exercise β-amyloid (*p* < 0.05), change in β-amyloid (*p* < 0.05), brain-derived neurotrophic factor (*p* < 0.01), and cognitive function (*p* < 0.05). **Conclusions**: Therefore, it is suggested that the circuit training used in this study could be an effective exercise method for improving the risk factors of cognitive impairment in obese elderly Korean women.

## 1. Introduction

The global average life expectancy has been increasing, leading to a rapid progression of aging worldwide, and the prevalence of dementia is rapidly increasing worldwide [1]. Dementia is the loss of cognitive functioning and ability to lead one’s daily life. It is the most prevalent neurodegenerative disease among the elderly, and such cognitive dysfunction is a primary cause of poor quality of life [2]. The types of dementia include Alzheimer’s disease (AD), vascular dementia, Parkinson’s disease, and other types of dementia caused by various underlying factors. AD, which accounts for approximately 70% of dementia cases [3], causes dysfunction of the synapses as nerve cells die [4] due to the deposition of β-amyloid (Aβ) [5] and reduction in brain-derived neurotrophic factor (BDNF) [6]. It negatively affects cognitive and neurodegenerative processes and causes a state of cognitive dysfunction in perception, logic, reasoning, and awareness [7].

According to a report by Seoul National University Hospital [8], the neuropathology of AD begins 15 to 20 years prior to the appearance of such symptoms as problems with memory and spatiotemporal abilities, loss of linguistic abilities, and loss of identity, and the decline in cognitive function begins in the 50s and accelerates after the age of 65 [9]. In addition, the prevalence of dementia is higher among females than males [3], and females aged 60 to 74 are at a higher risk of cognitive decline than females aged 50 to 59 [10]. Therefore, to prevent AD, it is important to manage the pathological factors that cause dementia, and the pathological risk factors include Aβ and BDNF [11].

Aβ is generated in the brain when the amyloid precursor protein (APP) is metabolized by β-secretase and γ-secretase, which are enzymes located in the hippocampus, the most crucial area of the brain for memory and cognitive function. Aβ then aggregates to form amyloid plaques in the brain [12]. The accumulated Aβ induces neuronal cell death through the formation of amyloid plaques [13], leading to oxidative damage, blood–brain barrier (BBB) dysfunction, and the development of neurofibrillary tangles in nerve fibers [14]. This, in turn, causes AD by impairing learning and memory formation abilities, disturbing the processing of new information, and causing behavioral dysfunctions [15].

In this respect, reducing or inhibiting Aβ has been proposed as a primary treatment method for AD, but most treatments depend on existing medications. However, it is possible to lower plasma Aβ concentrations through exercise [16], and the greater the amount of physical activity, the lower the Aβ concentration that has been observed [17].

BDNF concentration in the body, on the other hand, becomes lower with increasing age. Because its concentration in elderly females is lower than in elderly males, it serves as an indicator of memory and cognitive impairment in older females [18]. Thus, a decrease in plasma BDNF levels increases the probability of AD onset [6], but it is possible to delay the progression of dementia by increasing BDNF levels through exercise [19].

Cognitive function is critical in determining quality of life in old age, so managing cognitive decline is necessary to maintain and improve quality of life [20]. Regular exercise in old age can reduce the occurrence of dementia by up to 60%, while low levels of physical activity increase the prevalence of AD by 25% [21].

Regular exercise can promote cognitive function and act as a major factor in the prevention of AD by increasing the expression of proteins involved in neuroregeneration in the brain, such as BDNF, and decreasing the expression of Aβ, a key component of AD [22]. While exercise can maintain and improve cognitive function, social distancing, as well as limited operations and closures of exercise facilities due to COVID-19, have changed existing patterns of daily life. This has led to decreased time spent on physical activities [23] and lower rates of participation in regular exercise compared to the pre-pandemic period [24]. This decline in physical activities has reduced energy consumption, leading to an increase in the probability of sarcopenic obesity [25].

Furthermore, obesity causes functional and structural changes in the brain by reducing the expression of neurotrophic factors such as BDNF, which plays a vital role in neuroplasticity and neurogenesis, and by damaging cerebral vessels and the BBB, leading to reduced cognitive function and cognitive impairment [26]. Obese elderly individuals are less cognitively capable than elderly persons with normal body weight [27].

Those newly participating in or continuing physical exercises require easy accessibility in terms of time and location and the generation of internal motivation. Exercising with others is more effective than exercising alone, and combinations of two or more safe exercises are more effective in improving motor functions in the elderly than performing a single type of exercise [28]. Among physical exercises, circuit training using weight load alternates between aerobic and resistance exercises and allows for engaging in various exercises within a short period of time and continued participation with interest. Additionally, circuit training is an enjoyable exercise method for anyone because of its low risk of injury, limited spatial restrictions, and flexibility to be performed with a group rather than alone. Specifically designed to enhance muscle mass, body composition, functional capacity, muscle strength, and cardiovascular health, circuit training is well-suited for seniors, involving repetitive strength exercises with minimal rest between sets, offering a range of benefits [29].

Therefore, the present study aims to investigate the effect of circuit training on β-amyloid, BDNF, and cognitive function in untrained obese elderly Korean women aged 65 to 70, who are at a higher risk of developing dementia and accelerated cognitive decline than elderly males. It also aims to provide baseline data to be used in programs for preventing AD and improving physical functions and quality of life in the future.

## 2. Materials and Methods

### 2.1. Subjects

**Figure 1:**
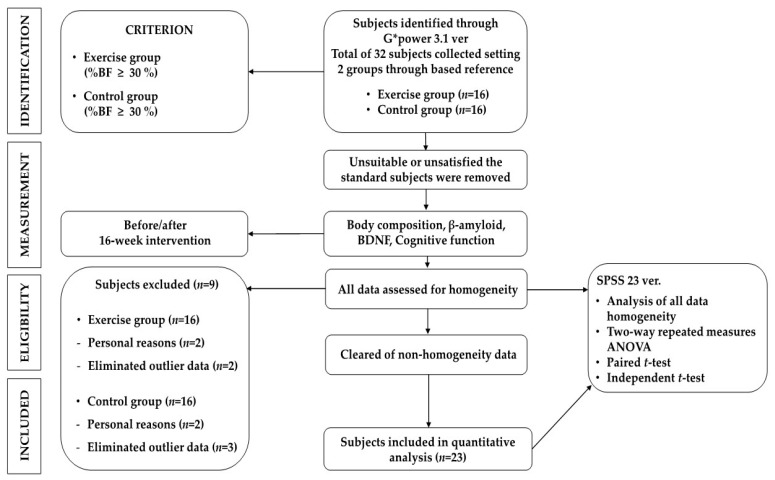
Procedures of this study.

Twenty-three obese elderly women (living in Busan, Republic of Korea) who did not exercise regularly (defined as <20 min of exercise twice a week) and had not taken drugs for obesity treatment in the previous six months were recruited for the current study. The participants were between the ages of 65 and 70 and had body fat percentages of 30% and over. Simple random sampling was used to assign 16 to the control group and 16 to the exercise group. Before the experiment, this study was approved by the Pusan National University Institutional Review Board (this study was a study derived from the PNU IRB/2017_68_HR), and the subjects were fully informed regarding the purpose of the study; only those who were willing to participate in the experiment submitted their consent for participation. The intervention period for all participants spanned 16 weeks, from early July to early November. Both the measurement and analysis of test results were conducted on 11 cases in the control group and 12 cases in the exercise group after excluding withdrawals from the experiment due to personal circumstances. The physical characteristics of the participants and the procedures of the study are shown in Table 1 and Figure 1.

### 2.2. Circuit Training Program

The exercise program was a modified and supplemented version of Ha et al.’s [29] program, consisting of 10 min of warm-up, 40 min of the main exercise, and 10 min of cool-down. The 60 min program was conducted three times a week for 16 weeks. This exercise routine included a variety of exercises such as standing low jump and shake, modified push-up, jogging in place, squats, high knee, crunch, step box, lunges, and jumping jacks.

**Figure 2:**
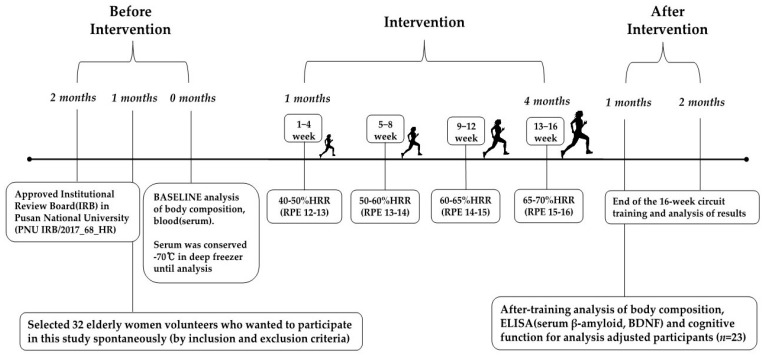
Flowchart of exercise intervention.

The target heart rate was checked using wireless heart rate monitors (Polar RS400sd, USA), and ratings of perceived exertion (RPE) were used to maintain the exercise intensity. Heart rate reserve (HRR) was gradually increased throughout the exercise period from 40% to 50% (RPE 12–13) for weeks 1 through 4, 50% to 60% (RPE 13–14) for weeks 5 through 8, 60% to 65% (RPE 14–15) for weeks 9 through 12, and 65% to 70% (RPE 15–16) for weeks 13 through 16 to ensure the safety of the participants [30]. The progression of the circuit exercises is shown in Figure 2.

### 2.3. Measurement Items and Analytical Method

#### 2.3.1. Body Composition

The examinations were carried out by the same experienced examiner (researchers) for all measurements and analyses. For the measurement of body composition, height was measured using a DS-103 automatic extensometer (Jenix Co., Seoul, Republic of Korea) after all metals participants were carrying on their body had been removed, and weight (kg), body fat mass (kg), skeletal muscle mass (kg), and body fat percentage (%) were measured using an Inbody 770 body composition analyzer (Biospace Co., Seoul, Republic of Korea). Before undergoing Inbody measurements, subjects refrained from participating in moderate- to high-intensity exercise for 12 h. Additionally, they abstained from consuming any food or beverages for 4 h.

#### 2.3.2. Blood Analysis

All pre- and post-experiment tests for measuring β-amyloid, brain-derived neurotrophic factor (BDNF), and cognitive function were performed under the same conditions using the same method. The participants received instructions from a clinical pathologist to maintain a fasting state from 8 PM on the day before the test to 8–10 AM on the day of the test.

Medical technologists collected 10 mL of blood from the antebrachial vein using vacutainer tubes and single-use syringes. The collected blood was put in a serum-separating tube (SST) and separated at 3000 rpm for 20 min using a Combi-514R centrifuge (Hanil, Gimpo, Republic of Korea). After the serum was separated, the supernatant was transferred to a 1.5 mL microtube and stored at −70 °C for analysis.

An enzyme-linked immunosorbent assay (ELISA) technique was employed for the analysis of Aβ using a human amyloid beta (aa1-42) Quantikine ELISA Kit (R&D Systems, Minneapolis, MN, USA) and a Thermo Scientific Multiskan Go spectrophotometer (Thermo Scientific, Waltham, MA, USA).

BDNF was also analyzed using the ELISA method with a Total BDNF Quantikine ELISA Kit (R&D Systems, Minneapolis, MN, USA) and a Thermo Scientific Multiskan Go spectrophotometer (Thermo Scientific, Waltham, MA, USA).

#### 2.3.3. Cognitive Function

Cognitive function was measured using the Mini-Mental State Examination—Korean (MMSE-K), the modified and supplemented version of the Mini-Mental State Examination for the elderly in Korea. The MMSE-K has a total of 12 items and a total score of 30. Scores of 24 and above indicate definitive normal, 20 to 23 suspected dementia, and 19 and below definitive dementia. The examination was conducted through one-on-one interviews, and the total score was calculated by adding the score of each item. The study was conducted on normal individuals who did not have suspected or confirmed dementia, which is why all 23 participants scored 24 points or higher.

#### 2.3.4. Data Analysis

Regarding descriptive statistics, the means (M) and standard deviations (SD) were calculated for each measurement item using SPSS ver.23.0 (SPSS Inc., Chicago, IL, USA). The inter-group heterogeneity of the variables was tested using Levene’s F-test. The mean difference tests for comparison before and after 16 weeks within each group were conducted using a paired *t*-test, the between-group differences were tested using an independent *t*-test, and the time interactions using a two-way ANOVA. The significance level was 0.05 for all statistical tests. The sample size in this study was estimated to be *n* = 23 using G-Power 3.1 (Universitat Dusseldorf, Dusseldorf, Germany), based on the repeated measures analysis of variance and under the following conditions: effect size = 0.5, α = 0.05, and power = 0.80.

## 3. Results

### 3.1. β-Amyloid

**Figure 3:**
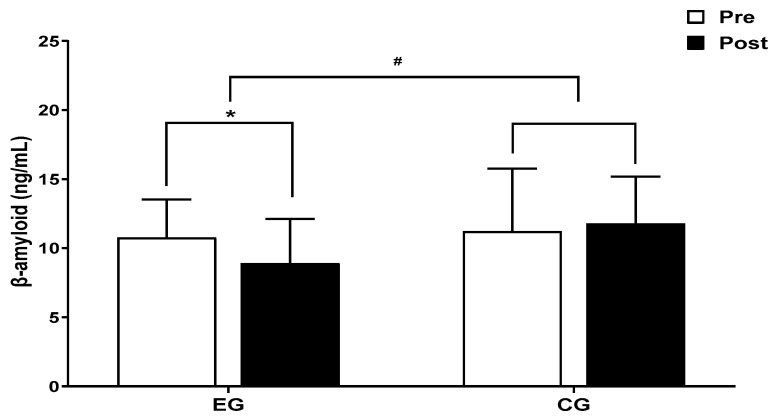
Comparisons of β-amyloid after 16-week circuit training. * *p* < 0.05: pre vs. post; # *p* < 0.05: exercise vs. control.

**Figure 4:**
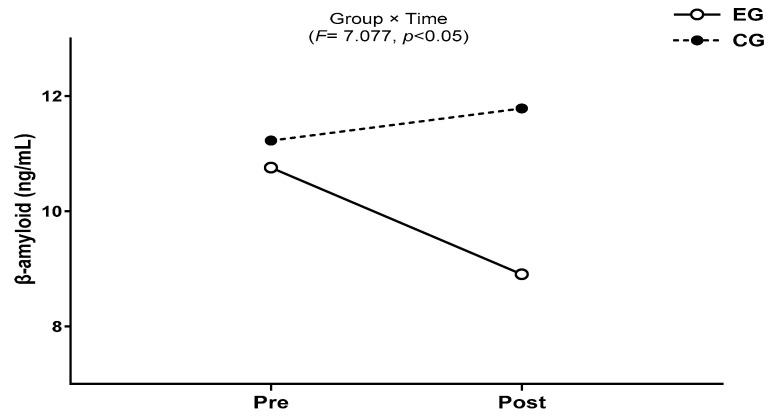
Result of two-way repeated measures ANOVA of β-amyloid after 16-week circuit training.

The results for the intra-group and inter-group analysis of changes and interactions of β-amyloid are shown in Figure 3 and Figure 4.

A significant group × time interaction was found (*F* = 7.077, *p* < 0.05). The exercise group showed a significant decrease in β-amyloid over time (*t* = −3.086, *p* < 0.05), and the inter-group comparison showed a significant difference after post-exercise values (*t* = 2.902, *p* < 0.05), and amount of change (*t* = 2.660, *p* < 0.05).

### 3.2. BDNF

**Figure 5:**
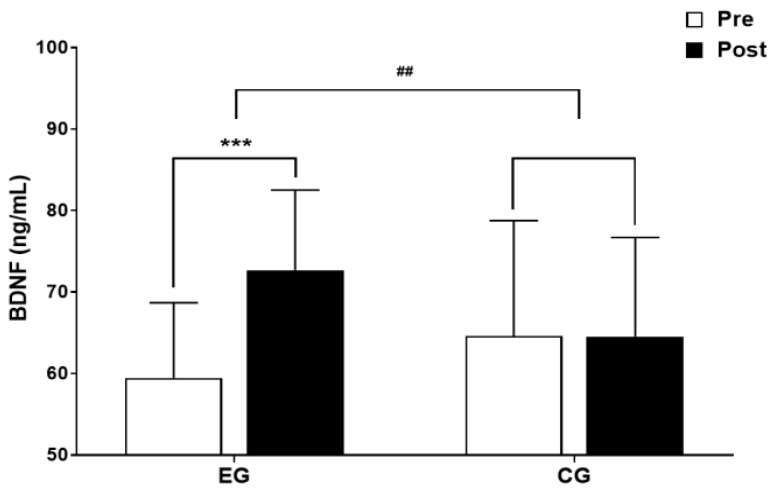
Comparisons of BDNF after 16-week circuit training. *** *p* < 0.001: pre vs. post; ## *p* < 0.01: exercise vs. control.

**Figure 6:**
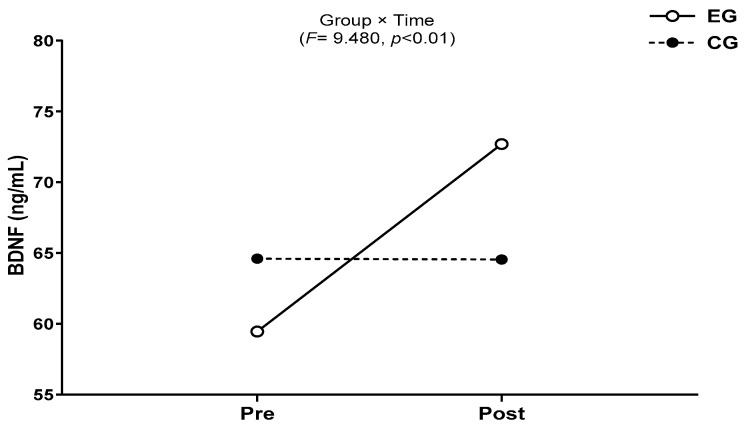
Result of two-way repeated measures ANOVA of BDNF after 16-week circuit training.

The results for intra-group and inter-group analysis of changes and interactions of BDNF are shown in Figure 5 and Figure 6.

A significant group × time interaction was found (*F* = 9.654, *p* < 0.01). The exercise group showed a significant increase in BDNF over time (*t* = 4.910, *p* < 0.001), and the inter-group comparison showed a significant difference in the amount of change (*t* = −3.107, *p* < 0.01).

### 3.3. Cognitive Function

**Figure 7:**
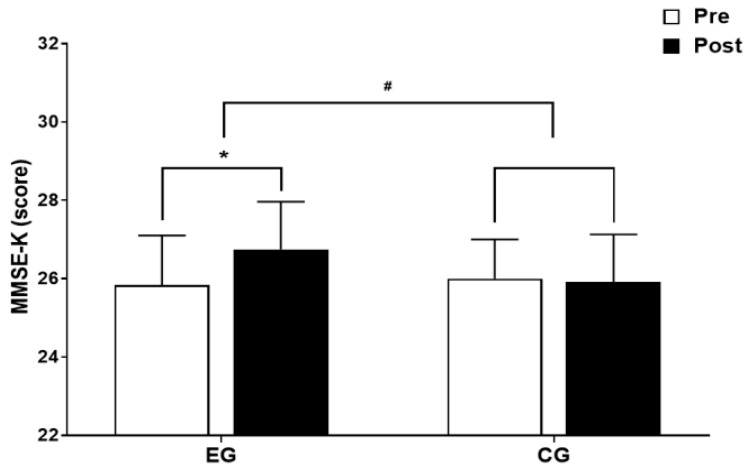
Comparisons of MMSE-K after 16-week circuit training. * *p* < 0.05: pre vs. post; # *p* < 0.05: exercise vs. control.

**Figure 8:**
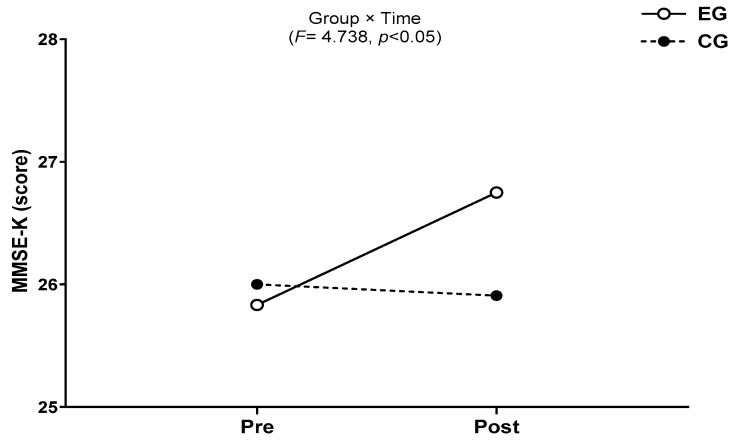
Result of two-way repeated measures ANOVA of MMSE-K after 16-week circuit training.

The results for intra-group and inter-group analysis of changes and interactions of cognitive function are shown in Figure 7 and Figure 8.

A significant group × time interaction was found (*F* = 4.738, *p* < 0.05). The exercise group showed a significant increase in cognitive function over time (*t* = 2.727, *p* < 0.05), and the inter-group comparison showed a significant difference in the amount of change (*t* = −2.177, *p* < 0.05).

## 4. Discussion

Our findings indicate that regular and continuous circuit training is effective in improving Aβ, BDNF, and cognitive function in obese elderly Korean women. Taken together, our results indicate that the circuit training with sequential aerobic and anaerobic exercises conducted in the present study can be an effective method for preventing dementia by improving the levels of Aβ and BDNF, which are risk factors for cognitive impairment in obese elderly females, as well as hindering cognitive decline.

Because hippocampal neurons are produced even after a person has reached adulthood [31], long-term exercises of appropriate intensity have a positive effect on preventing AD [32] and improving cognitive function through enhancing cognitive performance, short-term memory, and long-term memory [33]. Therefore, exercise in the elderly at the onset of aging can improve cognitive function by affecting the reduction in Aβ and increase in BDNF, which are risk factors for AD [22].

Aβ decreased in elderly females after the intervention of 60 min sessions of aerobic and anaerobic exercises three times a week for 12 weeks [22]. A decrease in Aβ was also observed in elderly females who engaged in aquarobics exercises for 50 min per session three times a week for 24 weeks [34], as well as in elderly females who performed a combination of elastic band and aquarobics exercises for 90 min per session three times a week for 16 weeks [16].

Prior studies on Aβ and exercise intensity in elderly females reported a significant decrease in Aβ after participants performed Taekwondo Poomsae at 50–75% HRR for 60 min per session three times a week for 12 weeks [35], a decrease in the Aβ index after participants performed a combination of physical stimulation and walking exercises at 60–70% of maximum heart rate for 45–60 min per session for 24 weeks [36], and a decrease in Aβ after participants performed combined aerobic and anaerobic exercises at 50–70% intensity of target heart rate for 60 min per session three times a week for 12 weeks [22].

The mechanism of Aβ decrease can generally be explained as follows: α-secretase metabolizes APP when blood flow to the brain is increased during exercise and inhibits the formation of plaques in the brain [37]. Therefore, the reason for the decrease in Aβ in the present study appears to be the choice of 40–70% HRR as an appropriate exercise intensity for elderly females whose overall strength has been reduced due to aging. Regular circuit training increased the blood flow to and stable oxygenation of the brain, causing a rise in BDNF concentration, which is essential for the survival of nerve cells. The increased BDNF concentration activated non-amyloidogenic pathways with α-secretase and γ-secretase, leading to enhanced amyloid metabolism and a decrease in Aβ.

Serum and plasma BDNF in the circulation can be indicators of BDNF in the central nervous system because BDNF is a myokine that is released by skeletal muscle cells and can cross the BBB in both directions [38], and the elevated plasma BDNF concentration is influenced by the type and intensity of exercise [39].

Prior studies on BDNF expression according to types of exercises showed that, for myokinetic BDNF, muscular resistance exercises that directly stimulate muscles could increase plasma BDNF concentration [40]. However, aerobic exercises were more effective than resistance exercises [41], and concurrent exercises were more effective than single resistance exercises in increasing plasma BDNF concentration [42]. As BDNF is affected by exercise intensity, the increase in BDNF concentration was higher for mid- and high-intensity exercises (70% oxygen uptake reserve; %VO_2_R) than for low-intensity exercises (40% VO_2_R) [43], and only exercising with an intensity of 60% HRR or higher makes that increase possible [44]. Choi and Yoon [45] reported a significant increase in BDNF as a result of performing concurrent exercises of RPE 11–15.

Therefore, the increase in BDNF in the current study is most likely due to the application of aerobic-type circuit exercises with 65–70% intensity, which could provide more nutrition and oxygen to the brain cells by increasing the flow of blood to the brain and its speed. In addition, resistance exercises involving repeated contractions of skeletal muscles are likely more effective than a single movement.

The general characteristics of BDNF changes are a decrease in plasma BDNF levels with increased age [46], lower levels for ill or diseased persons [47], and lower levels in females than in males [18]. However, adults without existing medical conditions who have stable BDNF concentrations can no longer significantly increase their BDNF concentrations due to the ceiling effect [48]. Additionally, reduced body fat through exercise increases the rate of BDNF expression in obese persons [49], and the sensitivity of BDNF change is higher in obese females than in obese males [50].

Therefore, the higher sensitivity to BDNF of the participants, who were obese elderly females, and the improvement in body fat percentage from positive changes in body composition through exercise are suspected to be additional factors explaining the significant increase in BDNF concentration in the current study.

Cognitive function is enhanced by regenerating damaged neurons and peripheral nerves and generating cerebral neurons through the expression of neurotropic factors [51,52]. The MMSE-K index improved in elderly females after they performed 60 min of circuit exercises three times a week for 12 weeks [29], and the MMSE-K score increased from 25.14 to 26.86 after 12 weeks of concurrent exercise among women with a mean age of 65 [53]. In addition, an improvement in physical and psychological abilities was observed after participation in the exercises; as a result of performing combination exercises including 15 to 30 min walks, strength exercises, and aquarobics exercises three or more times per week, elderly persons aged 65 and above showed enhanced memory, problem-solving abilities, linguistic abilities, attention, concentration, and mind and body stability [54].

Exercise must be of an appropriate duration, intensity, and frequency to improve cognitive function [55], and cognitive decline cannot be prevented when the duration and intensity of exercises are reduced [56]. An analysis of cognitive function and the duration of exercise intervention showed that the effect was prominent in the order of 16 weeks or more, 12 weeks, 8 weeks, and 4 weeks, with the most significant effect of exercise observed for 16 weeks, followed by 12 weeks [57,58]. Mid- and high-intensity physical activities correlated highly with cognitive function [59]. The group that participated the least in mid- and high-intensity exercises was 0.65 times more likely to be at risk of cognitive dysfunction than the group that participated the most [60]. Those who were more active, especially those who exercised more than three times a week, were at a lower risk of developing dementia [61].

The results of these prior studies collectively show that exercises are effective in maintaining cognitive function or slowing cognitive decline only when mid- and high-intensity exercises are performed more than three times a week for 16 weeks. Therefore, the improvement in cognitive function in the current study seems to have resulted from continuous and regular exercise participation three times a week for 16 weeks, while increasing HRR and RPE every four weeks to mid- to high-intensity exercise with 65–70% HRR during the final weeks. The expression of BDNF, a myokine released during the contraction of skeletal muscles, is increased due to repeated muscle contraction in the upper and lower extremities, and cognitive function is enhanced by the mechanism of inhibiting the α-secretase activity in the non-amyloidogenic pathway that results in the production of Aβ, which causes toxicity.

People who have been physically active or participated in regular exercise programs before becoming elderly delay cognitive decline, have lower Aβ expression, are at less risk of cognitive impairment when elderly [62], and have increased BDNF [63] compared to those who have not. A 10-year follow-up study of 295 males revealed that the cognitive function of the group of elderly persons with reduced physical activity decreased 2.6 times more than the group that maintained physical activity, and a delay in cognitive decline was only possible when engaging in exercises of moderate intensity or higher [56]. An analysis of the relationship between walking and cognitive function among women aged 65 years or older showed that the group that walked effectively prevented the decline of cognitive function after six to eight years [64].

Several limitations of the present study need acknowledgment. Firstly, the assessment of cognitive function, although conducted by an expert, faced challenges in deriving the exact mechanisms for improving cognitive function due to the simplicity of the examination and the reliance on scores determined by a single summation. It is recommended that future studies adopt a comprehensive approach to measuring cognitive function, combining both questionnaires and imaging tests. Furthermore, it is deemed necessary to validate the cognitive function improvement effects of circuit training in subjects with cognitive impairment in future studies. Secondly, considering the current study’s focus on obese elderly females with a restricted 16-week exercise program, subsequent research should include comparative studies involving males and females, individuals with varying body weights, and different program durations to provide a more comprehensive understanding. Lastly, this study did not account for the potential impacts of cerebrovascular diseases, such as brain damage or stroke, in obese individuals. Therefore, future research should encompass a broader range of studies that consider cardiovascular risk factors.

## 5. Conclusions

Therefore, when elderly females begin to age, they must exercise regularly and continuously to avoid an increase in Aβ and a decrease in BDNF, risk factors of AD, thereby preventing cognitive decline and maintaining and improving their quality of life.

## Figures and Tables

**Table 1:** Changes in body composition after 16 weeks of circuit training.

	CG (*n* = 11)		EG (*n* = 12)	
	Pre	Post	Pre	Post
Age (years)	67.97 ± 1.73		66.97 ± 1.63	
Height (cm)	154.73 ± 3.69		152.55 ± 4.19	
Weight (kg)	55.49 ± 4.44	55.75 ± 4.72	57.33 ± 6.61	57.24 ± 6.59
BMI (kg/m^2^)	24.45 ± 1.70	24.58 ± 2.00	24.67 ± 2.38	24.62 ± 2.31
SMM (kg/m^2^)	19.09 ± 1.40	18.99 ± 1.34	19.68 ± 1.85	19.98 ± 1.87 **
BFM (kg/m^2^)	19.82 ± 2.79	19.95 ± 2.71	20.35 ± 4.12	19.79 ± 3.96
%BF (%)	35.55 ± 3.12	35.65 ± 2.45	35.37 ± 3.31	34.33 ± 3.45 **

Values are *M* ± *SD*; BMI: body mass index, SMM: skeletal muscle mass, BFM: body fat mass, %BF: percentage of body fat, CG: control group, EG: exercise group, ** *p* < 0.01: pre vs. post intra-group.

## Data Availability

Data available on request due to restrictions.
